# The Rotavirus NSP4 Viroporin Domain is a Calcium-conducting Ion Channel

**DOI:** 10.1038/srep43487

**Published:** 2017-03-03

**Authors:** Thieng Pham, Jacob L. Perry, Timothy L. Dosey, Anne H. Delcour, Joseph M. Hyser

**Affiliations:** 1 Department of Biology and Biochemistry, University of Houston, Houston, TX, USA; 2 Alkek Center for Metagenomic and Microbiome Research, Department of Molecular Virology and Microbiology, Baylor College of Medicine, Houston, TX, USA; 3 Verna and Marrs McLean Department of Biochemistry and Molecular Biology, Baylor College of Medicine, Houston, TX, USA

## Abstract

Viroporins are small virus-encoded ion channel proteins. Most viroporins are monovalent selective cation channels, with few showing the ability to conduct divalent cations, like calcium (Ca^2+^). Nevertheless, some viroporins are known to disrupt host cell Ca^2+^ homeostasis, which is critical for virus replication and pathogenesis. Rotavirus nonstructural protein 4 (NSP4) is an endoplasmic reticulum transmembrane glycoprotein that has a viroporin domain (VPD), and NSP4 viroporin activity elevates cytosolic Ca^2+^ in mammalian cells. The goal of this study was to demonstrate that the NSP4 VPD forms an ion channel and determine whether the channel can conduct Ca^2+^. Using planar lipid bilayer and liposome patch clamp electrophysiology, we show that a synthetic peptide of the NSP4 VPD has ion channel activity. The NSP4 VPD was selective for cations over anions and channel activity was observed to have both well-defined “square top” openings as well as fast current fluctuations, similar to other viroporins. Importantly, the NSP4 VPD showed similar conductance of divalent cations (Ca^2+^ and Ba^2+^) as monovalent cations (K^+^), but a viroporin defective mutant lacked Ca^2+^ conductivity. These data demonstrate that the NSP4 VPD is a Ca^2+^-conducting viroporin and establish the mechanism by which NSP4 disturbs host cell Ca^2+^ homeostasis.

Ion channels are part of the fundamental cellular machinery used by cells to transduce signals across cellular membranes. As obligate cellular parasites, viruses too express ion channels to subvert cellular conditions in favor of virus replication and contribute to pathogenesis. Virus-encoded ion channels are called viroporins. Thus far, viroporins have been predominantly identified in animal viruses and are characterized as monovalent cation selective channels, with varying degrees of selectivity of potassium (K^+^) and sodium (Na^+^) over chloride (Cl^−^)^1^,^2^,^3^. Over the years, many viroporins have been studied using classical electrophysiological techniques, primarily by reconstituting synthetic peptides or purified recombinant protein of the viroporin into planar lipid bilayers. With few exceptions, viroporins show low selectivity between K^+^ and Na^+^, but generally show strong selectivity of monovalent cations over divalent cations like calcium (Ca^2+^)^4^. Thus, while most viroporins are initially targeted to the endoplasmic reticulum (ER), presumably their low permeability for Ca^2+^ prevents depletion of ER Ca^2+^ stores. However, a small number of viroporins are retained in the ER membrane and significantly deplete ER Ca^2+^ levels, suggesting that these viroporins target ER Ca^2+^ stores. Rotavirus (RV) NSP4 and picornavirus 2B proteins are the two best-characterized viroporins for the disruption of cellular Ca^2+^ homeostasis^1^.

RV is one of the leading causes of viral gastroenteritis in children, and despite multiple effective vaccines, RV kills 215,000 children under 5 years of age each year^5^. A hallmark of RV infection is a global elevation in cytosolic Ca^2+^ ([Ca^2+^]c) resulting from an increase in Ca^2+^ leak from the ER and increased Ca^2+^ influx through the plasma membrane (PM). Elevated [Ca^2+^]c is required for multiple processes of RV replication, including activation of autophagy, nucleation of RV replication complexes called viroplasms, and assembly of the outer capsid protein VP7^6^,^7^,^8^. We recently showed^9^ that the RV-mediated depletion of ER Ca^2+^ activates an ER Ca^2+^ sensor protein called stromal interaction molecule 1 (STIM1) and STIM1 in turn induces Ca^2+^ influx through store-operated calcium entry (SOCE), a homeostatic cellular mechanism to maintain ER Ca^2+^ levels. We further found that NSP4 viroporin activity is sufficient to activate STIM1 but NSP4 mutants that are deficient in viroporin activity neither activate STIM1 nor induce Ca^2+^ influx through SOCE^9^. Since STIM1 activation is caused by depletion of ER Ca^2+^, these data confirmed that NSP4 viroporin activity is responsible for the loss of ER Ca^2+^ in RV-infected cells and is the trigger for elevating [Ca^2+^]c, which is critical for RV replication.

Our previously proposed model, in which NSP4 viroporin activity depletes ER Ca^2+^, implies that the NSP4 viroporin domain forms a pore or ion channel that can conduct Ca^2+^. The purpose of this study was to first demonstrate that the NSP4 viroporin domain is able to form a *bona fide* ion channel and to determine whether NSP4 viroporin domain ion channels can conduct Ca^2+^ ions. Using both lipid bilayer and patch clamp electrophysiology techniques, we have confirmed that NSP4 is a cation-selective ion channel that can conduct both monovalent and divalent cations, including Ca^2+^ and Ba^2+^.

## Materials and Methods

### NSP4 Synthetic Peptides

**Figure 1:**
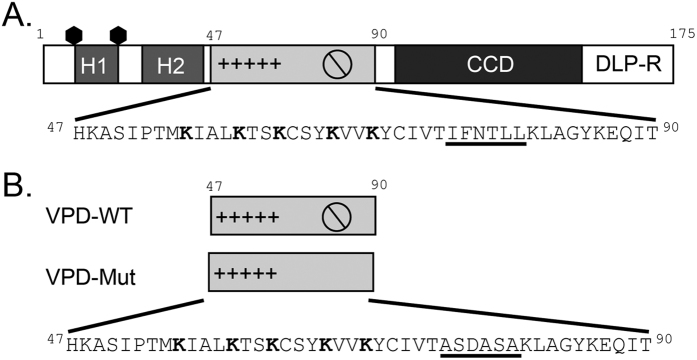
Illustration of NSP4 and VPD peptides. (**A**) Linear schematic of NSP4 and primary sequence of the viroporin domain (VPD, aa47–90) highlighting the pentalysine domain (+++++) with five conserved lysines (bold) and the amphipathic domain (∅) with the residues targeted by the VPD-Mut mutation (underlined). H1 and H2, hydrophobic domains 1 and 2, respectively; CCD, coiled-coil domain; DLP-R, double-layered particle receptor domain. The hexagon symbols represent the two N-linked glycosylation sites (aa8, 18). (**B**) Linear schematic of the two VPD peptides corresponding to wild-type (VPD-WT) or viroporin deficient (VPD-Mut) sequences. The primary sequence of the VPD-Mut peptide details the mutations (underlined) in the amphipathic domain.

The NSP4 short peptide construct encompassing the viroporin domain (VPD, residues 47–90) was designed (Fig. 1) and synthetic peptides of the wildtype (VPD-WT) and a mutant (VPD-Mut) were provided by LifeTein LLC. Peptides were provided lyophilized in aliquots of either 0.1 mg or 1 mg stored at −20 °C. Peptides were dissolved in either dimethyl sulfoxide (DMSO) solvent or water to achieve a stock concentration of 1–2 mg/ml. The DMSO-dissolved peptide was used within a week and stored at 4 °C, and water-dissolved peptide was stored at room temperature (RT). Water-dissolved samples stored at RT remained active for several months and reconstituted just as well as freshly dissolved peptide in DMSO.

### Chemicals

Asolectin (1,2-Diacyl-*sn*-glycero-2-phosphocholine Type II-S) was purchased from Sigma (Missouri, USA). All salts (KCl, MgCl_2_, CaCl_2_, BaCl_2_, NaCl), Hepes, MOPS, K-EDTA, and sucrose were also obtained from Sigma with at least >99.0% purity. Hexadecane was obtained at TCI (Oregon, USA) and pentane (high purity solvent grade) from Burdik & Jackson (New Jersey, USA). DMSO was obtained from EMD Millipore (Massachusetts, USA). For giant unilamellar liposomes, 1,2-diphytanoyl-sn-glycero-3-phosphocholine (DPhPC) and cholesterol were purchased from Avanti Polar Lipids (Alabama, USA).

### Planar Lipid Bilayer Electrophysiology

The planar lipid bilayer electrophysiological technique used a Teflon chamber (made in-house at the University of Houston) where the two halves are separated by a 0.01 mm-thick polytetrafluoroethylene (PTFE, Teflon) film (Goodfellows). Within the center of the Teflon film, an aperture of approximately 100 μm in diameter was created utilizing a High-Frequency Spark Tester PPM MK3 (Buckleys Ltd.) adjusted to 45 kV. In order to create a hydrophobic interface to attract lipids, the aperture was treated with a 1% hexadecane in pentane solution. One and a half mL of buffer T (1 M KCl, 5 mM Hepes, pH 7.2) or PA (150 mM KCl, 5 mM Hepes, pH 7.2) was added to each half of the chamber. Five microliters of asolectin dissolved in pentane at a concentration of 5 mg/mL was added to the surface of the buffer on both sides. Ag/AgCl electrodes were then inserted into the buffer solutions. The chamber-electrode configuration was then allowed to equilibrate before formation of a lipid bilayer over the aperture. A membrane bilayer was formed over the aperture by lowering and raising the buffer level in one of the half-chambers and monitoring the resistance on the oscilloscope. A high resistance indicated that a bilayer had formed. A transmembrane voltage of + or −90 mV was then applied to check membrane stability for 10 minutes. This was also performed to ensure that there was no contaminating channel-like activity prior to the addition of channel-forming protein. If the stability test showed no contaminants and a stable baseline, then NSP4 peptide dissolved in DMSO or water (4.0–8.0 μg) was added to one half-chamber, defined as the *cis* side. A membrane potential of + or −90 mV and stirring of the *cis* side were typically used to promote insertion. Channel insertion was detected by the appearance of channel activity. Once channel activity was detected, stirring was stopped to limit further insertions.

### Proteoliposome Reconstitution for Patch-clamp

A dehydration-rehydration method was used to reconstitute NSP4-VPD into asolectin liposomes as described^10^. NSP4-VPD was dissolved in either DMSO or water at a concentration of 2 mg/mL, and added to giant multi-lamellar liposomes at a protein:lipid ratio of 1:2000 (w:w), prior to the centrifugation and dehydration steps.

An alternative technique for liposome reconstitution (hereafter referred to as “the cloud method”) was also performed^11^ and modified for our use. Asolectin was dissolved in chloroform at a stock concentration of 10 mg/mL. Two hundred μL of this stock solution were added to a 0.5 mL DRAM glass vial and dried under a nitrogen stream to form a film along the inner walls of the DRAM. Liposomes were then allowed to swell into a 1 mL solution of 0.4 M sucrose in water while incubated at 45 °C on an orbital water-bath shaker at 150 RPM for 3 hours. Once a cloud of liposomes had formed within the sucrose solution, protein was added at the desired protein:lipid ratio and further incubated overnight with shaking as above. The protein:lipid ratio ranged between 1:20 and 1:200 (w:w) using NSP4 dissolved in either DMSO or water at a stock concentration of 2 mg/mL. DRAMs were then removed from the orbital shaker and chilled at 4 °C for 2 hours in order to stiffen the liposomes for patch-clamp. Three microliters of liposomes were taken from the liposome cloud and added directly to the patch-clamp bath chamber for patch-clamp experiments.

### Traditional Patch-Clamp Electrophysiology

The pore-forming activity of NSP4 peptide was investigated with the traditional patch-clamp technique, as described^10^. Micropipettes of ~10 ΜΩ resistance were pulled from glass capillaries with a P87 pipette puller (Sutter Instruments) and filled with buffer A (150 mM KCl, 10 μM CaCl_2_, 0.1 mM EDTA, 5 mM Hepes, pH 7.2) or buffer BA (75 mM BaCl_2_, 5 mM Hepes, pH 7.2). Reconstituted proteoliposomes were incubated in buffer B (buffer A + 20 mM MgCl_2_) to induce swelling of unilamellar blisters from the liposomes for patch-clamp experiments. The pipette was immersed in the bath with constant positive pressure and micro-manipulated to contact the blister to achieve 1.0–10.0 GΩ seal. Patches were excised with brief exposure to the air and then re-immersed in the bath solution. This solution was then quickly exchanged for either buffer A, buffer BA, or buffer CA (75 mM CaCl_2_, 5 mM Hepes, pH 7.2). All potential offsets were canceled electronically in symmetric buffer A before data acquisition. The reference electrode (World Precision Instruments) was in contact with the bath solution via a 3 M KCl agar bridge. Control experiments performed on liposomes lacking reconstituted NSP4 showed no channel activity.

### Planar Lipid Bilayer and Traditional Patch-Clamp Data Acquisition and Analysis

Experiments with either the planar lipid bilayer or the traditional patch-clamp technique were performed under voltage-clamp (ground is the *trans* side in planar lipid bilayer, and the bath in patch-clamp). Currents were recorded with an Axopatch-1D amplifier (Axon Instruments) and a CV-4 or a CV-4B head-stage in the patch-clamp or planar lipid bilayer setup, respectively. For recordings up to 10 minutes, currents were filtered at 500 Hz through a low-pass Bessel filter and digitized at 1.25-ms sampling intervals (ITC-18, Instrutech). For shorter high-resolution recordings of 1–2 minutes, the currents were filtered at 1 kHz and sampled at 100 μs sampling intervals. Currents were digitized using the Acquire software (Bruxton), and analyzed with pClamp (Axon Instruments).

### Port-a-Patch Electrophysiological Recording of Giant Unilamellar Vesicle Bilayers

Giant Unilamellar Vesicles (GUVs) were generated by the electro-swelling method using the Vesicle Prep Pro machine (Nanion, Germany) according to the manufacturer’s instructions. GUVs were generated by applying 20 μL of 10 mM DPhPC + 10% cholesterol dissolved in chloroform onto the slide, allowed to air dry, and 1 M sorbitol was used for the external solution for GUV swelling. GUVs were collected and either left as protein-free GUVs (negative control) or reconstituted with 10 ng/mL of the NSP4-VPD peptide dissolved in water for 1 hr at room temperature. After reconstitution the GUVs were kept at 4 °C until use. For patch clamp of GUVs we used the Port-a-Patch system (Nanion) with 8–12 megaohm patching chips (~1 μm aperture). Briefly, patches were established in symmetric 650 mM KCl buffer by adding 5 μL of protein-free or NSP4-reconstituted GUVs to the chips under slight negative pressure (−10 to −40 mbar). Upon contact with the aperture, the GUV ruptures to form a bilayer with high seal resistance (>10 Gigaohm) that was very reproducible. Once seals were established, residual sorbitol was removed by exchanging the bath buffer 3 times with 20 μL of 650 mM KCl and potential offsets were canceled electronically before data acquisition. Relative selectivity between cations and anions was performed by running voltage steps from −100 mV to +100 mV initially in symmetric 650 mM KCl and then after bath buffer exchange with 65 mM KCl to generate a 1:10 asymmetric 650 mM KCl/65 mM KCl gradient across the bilayer. Patch clamp data were acquired using an EPC10 amplifier and PatchMaster software (HEKA, Germany). The measured open channel current for each voltage was plotted and a linear regression line fitted to determine the reversal potential. The expected K^+^and Cl^−^ reversal potentials were calculated using the Nernst equation and the relative permeability of K^+^to Cl^−^ was determined using the Goldman-Hodgkin-Katz equation^12^.

### *E. coli* Lysis Assay & Immunoblot

The viroporin-mediated *E. coli* lysis assay was performed as previously described^13^ with some modifications. Starter cultures of BL21(DE3)pLysS *E. coli* bearing expression plasmids for wild-type NSP4 VPD (VPD-WT) or the viroporin defective mutant (VPD-Mut) were grown overnight. The NSP4 VPD-WT and VPD-Mut constructs included a C-terminal StrepII tag for immunoblot analysis. Overnight cultures were diluted 1:100 into fresh LB media supplemented with 1% glucose, 100 μg/mL ampicillin, 35 μg/mL chloramphenicol, and grown until the optical density at 630 nm (OD_630_) was 0.4 to 0.6 (approximately 3 hrs). Then 1 mM IPTG was added to induce NSP4 expression or left uninduced as a negative control. OD_630_ measurements of each culture were taken before IPTG induction and at 10 min intervals after induction for 90 min. Additional samples were taken at 0, 10, 30, 60 and 90 min post-induction for immunoblot analysis as previously described^14^. Gel loading was normalized to the OD_630_, and for samples of cells that had lysed, the OD_630_ just prior to lysis was used for normalization. Antibodies used were mouse monoclonal anti-StrepII antibody and alkaline phosphatase-conjugated goat anti-mouse IgG were used for detection.

### Membrane Protein Fractionation

NSP4 membrane fractionation was performed similarly to our previous NSP4 viroporin study^13^. Briefly, BL21(DE3)pLysS cultures were grown to an OD_630_ of 0.5–0.6, protein expression was induced with 1 mM IPTG and cultured for 90 min. Cells were pelleted by centrifugation (21,000 × g, 15 min) and resuspended in 2.5 mL PBS (total protein fraction [T]). The sample was then sonicated in PBS using a probe sonicator with two 1 min intervals. Membranes were pelleted by centrifugation (21,000 × g, 15 min) and the supernatant was collected as the soluble fraction [S]. The membranes were resuspended in ice cold 100 mM sodium carbonate, incubated for 30 min on ice, and pelleted again by centrifugation (21,000 × g, 15 min). The supernatant was collected as the peripherial membrane protein fraction [P] and the pellet was solubilized in 1% SDS in PBS for the integral membrane protein fraction [I]. Equal buffer volumes were used to maintain the same relative protein concentration as that of the starting material. Equivalent amounts of each fraction were analyzed by SDS-PAGE, as above. Antibodies used were rabbit anti-NSP4(120–147) peptide antisera^14^ or mouse monoclonal anti-StrepII antibody and alkaline phosphatase-conjugated goat anti-rabbit or anti-mouse IgG were used for detection.

### Liposome Ca^2+^-Flux Assay

The defined lipids 1-palmitol-2-oleoyl-sn-glycerol-3-phosphoethanolamine, 1-palmitol-2-oleoyl-sn-glycerol-3-[phosphor-rac-(1-glycerol)], and 1-palmitol-2-oleoyl-sn-glycerol-3-phosphocholine were solubilized in chloroform and mixed in a 3:1:1 weight-ratio respectively and the solvent was then evaporated using argon gas. Dried 1 mg lipid-cakes were hydrated in 0.5 mL 25 mM Hepes pH 8, 150 mM NaCl, and 50 μM Fluo-5N pentapotassium salt buffer. The lipid suspension was then extruded through a polycarbonate membrane with 1 μM diameter pores. Fluo-5N that was not trapped inside the liposomes was removed by running the solution through a 3.5 mL PD midiTrap G-25 column equilibrated with a 25 mM Hepes pH 8, 150 mM NaCl buffer. Fractions were collected and tested for free Fluo-5N dye and only the fractions with dye trapped inside the liposomes were used for the assay. Liposomes with good dye incorporation were added to a 25 mM Hepes pH 8, 150 mM NaCl, 2 mM CaCl_2_ buffer. Aliquots of this final liposome solution were loaded onto a 96-well plate and a time course of fluorescent signals (494/518) were measured using a FlexStation 3 (Molecular Devices) before and after the addition of either 15 μM VPD-WT, 15 μM VPD-Mut, or vehicle (water).

## Results

Our previous studies mapped NSP4 viroporin activity to a 44-amino acid peptide corresponding to residues 47–90, and this region contains a cluster of basic residues and a predicted amphipathic α-helix, which are signature viroporin motifs^13^. Thus, we used synthetic peptides of this minimal NSP4 viroporin domain (VPD) (Fig. 1) to determine whether it has *bona fide* ion channel activity using both planar lipid bilayer and liposome patch clamp techniques.

### Electrophysiological Analysis of NSP4 VPD

**Figure 2:**
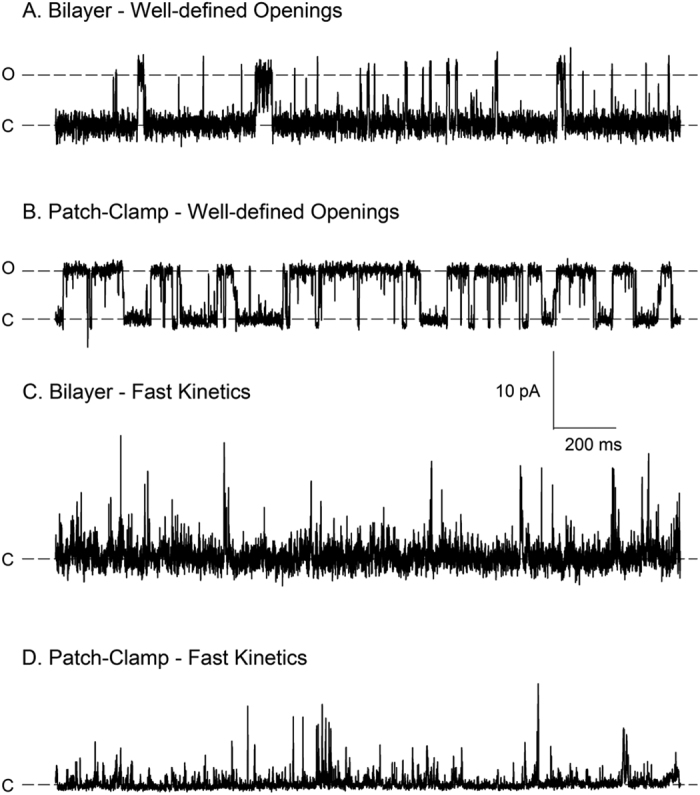
Electrophysiological signatures of VPD-WT in patch-clamp and planar lipid bilayer experiments. Representative traces of VPD-WT in either planar lipid bilayer or patch-clamp, as indicated, show defined opening events with discrete levels of conductance (**A** and **B**) or rapid current fluctuations (**C** and **D**). For planar lipid bilayer experiments, either 2.5 μg (**A**) or 12.5 μg (**C**) of VPD-WT dissolved in DMSO was added to the *cis* side of the bilayer. For patch-clamp experiments, VPD-WT dissolved in DMSO was reconstituted into multilamellar liposomes using the rehydration/dehydration method at either a 1:3000 (**B**) or 1:200 (**D**) protein-to-lipid ratio (w:w). Symmetric buffer A and buffer PA were used in the patch-clamp or planar lipid bilayer experiments shown, respectively. The applied voltage was +90 mV for all traces. The labels “c” and “o” denote the closed and open current levels, respectively. For panels **C** and **D**, discrete levels of conductance are not easily observed.

We tested the wild-type NSP4 VPD (VPD-WT) for ion channel activity using two well-established electrophysiology techniques, planar lipid bilayer and patch-clamp. In both techniques, we captured two unique electrophysiological signatures that characterize VPD-WT (Fig. 2). Figure 2A and B show that VPD-WT could display canonical well-defined opening events that measured ~65 pS in symmetric buffer conditions of 150 mM KCl when using either technique. These canonical “square-top” events are similar to those displayed by classical voltage or ligand-gated ion channels, with stochastic transitions between open and closed states of varying durations that averaged 10 and 25 ms, respectively. In addition, a second electrophysiological signature was observed for VPD-WT in a large majority of the experiments, and characterized by fast kinetics, as illustrated in Fig. 2C and D. Here, very transient and frequent fluctuations (open time <1 ms) to various open states give rise to a particularly “spiky” recording. Although this type of “spiky” ion channel activity is commonly observed among many viroporins^4^, conductance measurements require that the channel remain in the open state for a duration long enough to determine the current plateau with minimal error due to noise. When the opening transitions last for just a few sample points, it is challenging to make a reliable measurement of the open channel current. While it does provide evidence of ion channel activity, when the channel is in the fast kinetic state it is not possible to accurately determine the number of channels in the bilayer or patch or measure the single-channel conductance due to the transient nature of the openings. In addition, the high frequency of openings may be due to the concerted kinetics of multiple inserted VPD-WT oligomers in the lipid bilayer resulting in bursts of channel openings with very fast kinetics.

**Figure 3:**
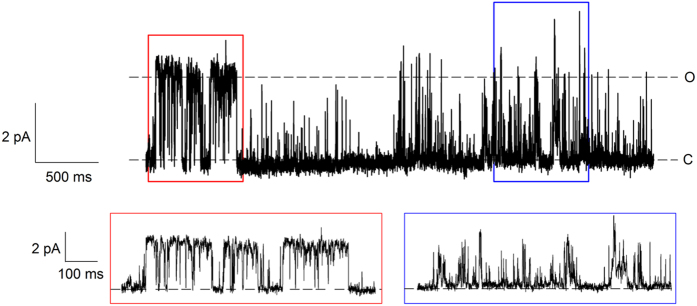
Shift in VPD-WT kinetics within the same recording. VPD-WT dissolved in DMSO was reconstituted into multilamellar liposomes at a 1:200 protein-to-lipid ratio (w:w) using the cloud method. The trace was obtained at +50 mV in patch-clamp in symmetric buffer A. The “c” denotes the closed level of conductance, and the “o” represents the open level of the channel obtained from well-defined openings. The red and blue boxes indicate the regions of the trace that are expanded below. The expanded segment in the red box has a conductance level of 50 pS. However, the expanded segment in the blue box has ill-defined conductance levels due to the fast channel kinetics.

On occasion, the two types of kinetic behaviors could be seen within the same recording, as shown in Fig. 3. A patch-clamp recording of VPD-WT dissolved in DMSO and reconstituted into liposomes utilizing the cloud method (see Materials and Methods) illustrates the ability of VPD-WT to shift kinetic states within the same experiment. As seen in the expanded segment (Fig. 3, red box), the reconstituted channel first displayed openings with a well-defined level of conductance measuring approximately 50 pS. However, within 500 milliseconds, the channel transitioned to a “spiky” behavior observed in a majority of the experiments (Fig. 3, blue box). While other viroporins have been characterized as having both fast current fluctuations (‘spiky’ activity) and well-defined openings, these two kinetic signatures have rarely (if ever) been demonstrated to occur during the same recording^4^. Further, recordings, such as in Fig. 3, show a clear transition between the kinetic state characterized by well-defined openings and that by fast kinetics. Since fast current fluctuations did not occur while the channel was already open to a well-defined conductance level, but rather stretches with fast and prolonged current transitions were consecutive, this recording originates from a single channel which oscillates between the two kinetics states, and not from multiple channels with different kinetic properties.

Altogether, we obtained 109 planar lipid bilayer recordings and 54 patch clamp recordings that showed that the VPD-WT peptide alone is capable of conducting monovalent ions. In ~70% of the recordings, VPD-WT displayed a discrete unitary level of conductance, as seen in canonical *bona fide* ion channels. In conditions of 150 mM KCl, the unitary conductance of VPD-WT ranged from 40 to 65 pS.

### Cation Selectivity of NSP4 VPD

**Figure 4:**
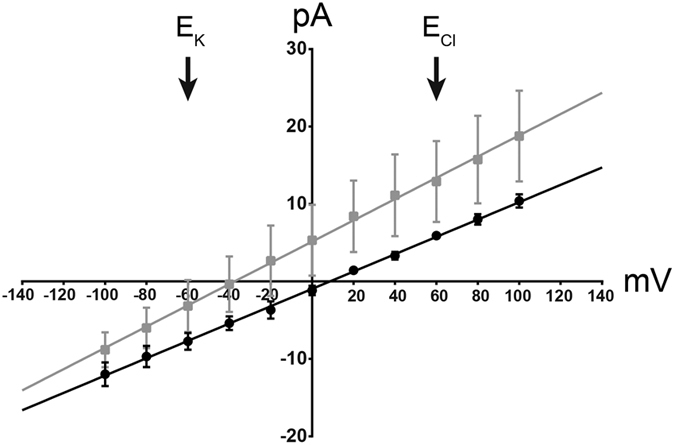
Cation selectivity of VPD-WT. VPD-WT dissolved in water was reconstituted into giant unilamellar liposomes used for patch clamp on a Port-a-Patch device to determine the current-voltage relationship in symmetric 650 mM KCl/650 mM KCl (black circles) or asymmetric 65 mM KCl/650 mM KCl (grey squares) buffers. The average single channel current (pA) is plotted for each voltage (mV) from −100 mV to +100 mV in 10 mV steps. The lines represent the linear regressions through the data points. The equilibrium potential for K^+^(E_K_) and Cl^−^ (E_Cl_) were calculated using the Nernst equation^12^ and are indicated (arrows). They represent the reversal potentials expected for a channel solely selective for K^+^or Cl^−^, respectively. The graph represents data from n = 3 liposomes (error bars represent standard deviation) from one preparation. The experiment was repeated with five separate liposome preparations with similar results.

Previously studied viroporins are generally cation selective with little conductivity of anions^4^. To determine the relative permeability of NSP4-VPD to cations and anions, we determined the current-voltage relationship in both symmetric and a 1:10 asymmetric KCl solutions. In symmetric 650 mM KCl, NSP4-VPD displayed a linear current-voltage relationship with a reversal potential of ~7 mV, representing a slight offset from the 0 mV value expected for symmetric buffers (Fig. 4). The bath buffer was then exchanged to 65 mM KCl to generate a 1:10 gradient across the patch. In this condition, the equilibrium potentials calculated with the Nernst equation are −58 mV for K^+^ (E_K_) and +58 mV for Cl^−^ (E_Cl_). Thus, the I/V plot of an ideal K^+^-selective channel would intersect the abscissa (0 pA) at −58 mV, while that of an ideal Cl^−^-selective channel would intersect the abscissa at + 58 mV. The intersect of the I/V plot with the abscissa is called the “reversal potential”. For NSP4 VPD, the 1:10 KCl gradient shifted the reversal potential to −39 mV, closer to E_K_ and away from E_Cl_. This demonstrated that NSP4-VPD selectively conducted cations (K^+^) over anions (Cl^−^). With this data, we used the Goldman-Hodgkin-Katz equation^12^ to determine that NSP4-VPD has a ~16-fold greater selectivity for cations over anions, making it a strongly cation selective viroporin.

### NSP4 VPD-WT Conducts Calcium and Barium

**Figure 5:**
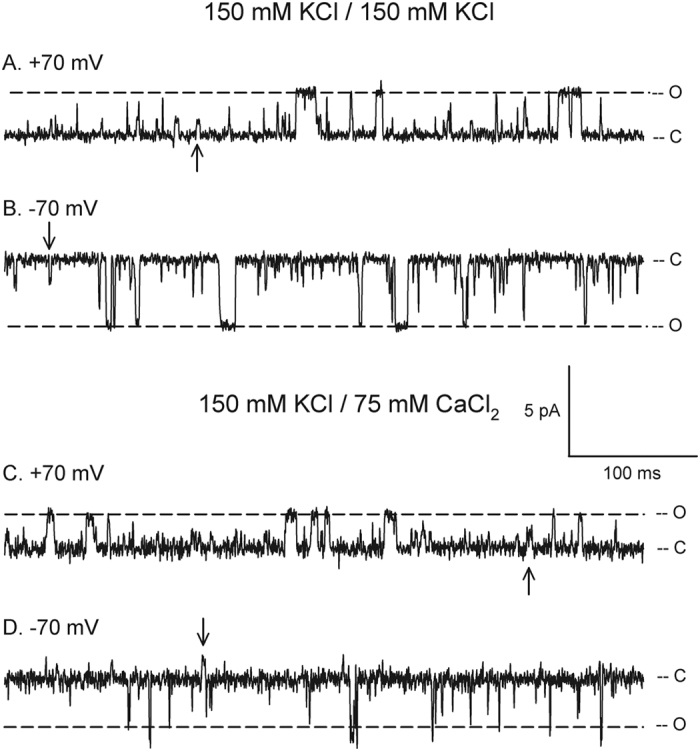
VPD-WT can conduct K^+^ and Ca^2+^ in patch-clamp experiments. The peptide was dissolved in DMSO and reconstituted with the dehydration/rehydration method at a protein:lipid ratio of 1:3000 (w:w). The traces were obtained at the indicated voltages in symmetric buffer A or asymmetric conditions of buffer A in the pipette and buffer CA in the bath, in the same patch. The conductance of the main state was 42 pS in symmetric buffer A (**A**,**B**), and 29 pS with buffer CA in the bath (**C,D**), as determined from I/V plots made from the well-defined openings observed in this experiment (Supplemental Fig. 1). The conductance of the substate (arrow) was 13 pS in both conditions. The labels “c” and “o” denote the closed and open current levels, respectively.

Given that VPD-WT is cation-selective and that a strong correlation has been established between NSP4 and elevation of cytoplasmic Ca^2+^, we predicted that NSP4 should be able to conduct Ca^2+^ ions. To test this, we recorded the activity of VPD-WT in patch-clamp in symmetric buffer A conditions [buffer A (containing 150 mM KCl, and 10 μM CaCl_2_) in both the bath and the pipette], and then perfused buffer CA (containing 75 mM CaCl_2_) into the bath. If the channel is able to conduct Ca^2+^, in these asymmetric buffer conditions opening transitions should be observed when a negative pipette voltage is applied to draw Ca^2+^ into the pipette. Figure 5 illustrates that this is indeed the case. At a pipette voltage of +70 mV or −70 mV, we observed well-defined levels of conductance measuring 42 pS in symmetric buffer A conditions (Fig. 5A,B). Occasionally a smaller 13 pS open state could also be observed (marked by the arrow in Fig. 5). When buffer CA was perfused into the bath, we continued to observe well-defined channel openings at both positive and negative voltages, indicating that the channel is indeed permeable to Ca^2+^, the sole cation able to sustain a current at negative pipette potentials (Fig. 5C,D). The conductance, however, was slightly reduced (29 pS vs 42 pS, Supp. Fig. 1), and the kinetics appear faster, but given the variability we observed in kinetic signatures, it is hard to ascertain whether this is due to the presence of Ca^2+^ or not. Current-voltage relationships obtained from these recordings (Supp. Fig. 1) showed that the reversal potential shifted only slightly (~7 mV) in asymmetric conditions. Therefore, the channel does not appear to be more selective for K^+^ than Ca^2+^.

**Figure 6:**
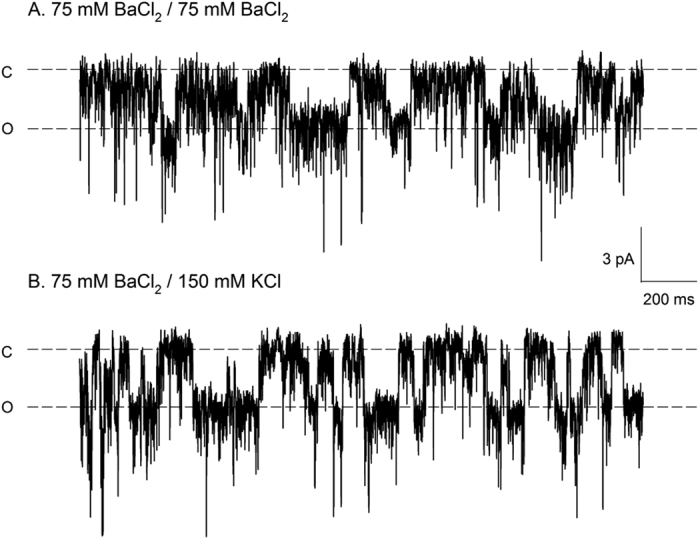
VPD-WT can conduct Ba^2+^. The peptide was dissolved in water and reconstituted with the cloud method at a protein:lipid ratio of 1:20 (w:w). The traces were obtained with patch-clamp in the indicated buffer conditions at −50 mV. The labels “c” and “o” denote the closed and open current levels, respectively. By convention, opening transitions are in the downward directions at negative voltages.

Many calcium channels can conduct Ba^2+^ ions^15^,^16^ often with a larger conductance than Ca^2+^. Therefore, we tested whether VPD-WT was also capable of sustaining Ba^2+^ currents, by introducing buffer BA into our patch-clamp configuration in symmetric conditions. Figure 6A illustrates that channel activity is indeed detectable in symmetric Ba^2+^ conditions, indicating that the channel can also conduct Ba^2+^ ions. The use of Ba^2+^ did not result in more patches with the well-defined openings, and fast current fluctuations were still ready observed (data not shown). We were able to determine a conductance of ~60 pS in symmetric 75 mM barium solutions from the well-defined openings observed in the traces. This value is similar to the conductance in 150 mM potassium solution (Fig. 6B) upon perfusion of buffer A on the same patch. Ba^2+^ conductance was larger than the conductance in 75 mM calcium solution, although the data was not obtained from the same patch due to patch rupture.

### Reconstitution Efficiency

Our initial dehydration/rehydration method yielded a relatively low reconstitution efficiency of ~10% (Table 1). Thinking that perhaps the synthetic peptide did not respond well to the dehydration step, we switched to the “cloud method” (see Materials and Methods) which might allow for slower and gentler reconstitution compared to the dehydration/rehydration method. Unfortunately, the cloud method did not improve the reconstitution efficiency (Table 1). We also found that the kinetics of the channel were largely unaffected by the reconstitution method. Patches with predominant fast kinetics or with well-defined openings either alone or in combination with fast transients were obtained from peptide reconstituted via either reconstitution method (Supp. Fig. 2).

In order to determine if the reconstitution technique affected the predominant kinetic state, we categorized our patches into 3 groups, according to the kinetic activity displayed throughout the experiment: “well-defined” openings only, “fast kinetics” only, and “mixed” (meaning the presence of both behaviors in the same experiment). Table 1 shows that, while more patches with only well-defined openings were obtained via the dehydration/rehydration method, more patches with the “mixed” activity were present using the cloud method. As a result, collectively, we observed well-defined openings in 64% of the *active* patches with the dehydration/rehydration method (25% “mixed” + 39% “well-defined”), but in 73% (46% “mixed” + 27% “well-defined”) of the *active* patches with the cloud method. Therefore, the cloud method might be a slightly superior method for reconstituting NSP4 VPD peptide over the dehydration/rehydration method.

### NSP4 VPD Mutant Lacks Ca^2+^Conductivity

**Figure 7:**
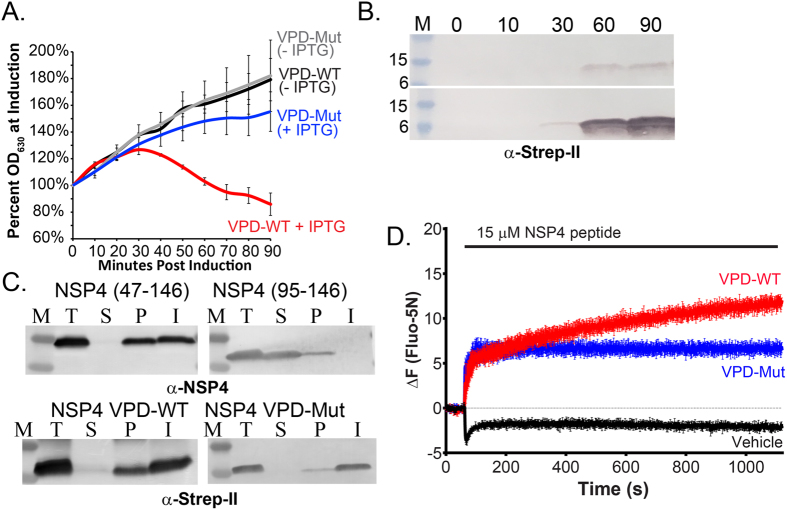
Viroporin-deficient VPD-Mut cannot conduct Ca^2+^. (**A**) Recombinant VPD-WT and VPD-Mut constructs were tested in the *E. coli* lysis assay. The optical density at 630 nm (OD_630_) of uninduced (-IPTG, black and grey lines) and VPD-WT (red line) or VPD-Mut (blue line) expressing cultures were determined at 10 min intervals for 90 min and presented as the percent OD_630_ relative to that at the time of induction. Traces are the average of 3 separate experiments and error bars represent the standard deviation. (**B**) Immunoblot of NSP4 expression for VPD-WT (top) and VPD-Mut (bottom) in the cultures measured in panel A. Samples for immunoblot were collected at the time post-induction indicated and detected using an anti-StrepII epitope antibody. Full-length blots are included in Supplementary Figure 3A. (**C**) Immunoblot analysis of bacterially-expressed NSP4 in total cell lysate [T], soluble proteins [S], peripheral membrane protein [P], and integral membrane protein [I] fractions. The upper panel shows NSP4 (47–146) and NSP4 (95–146) that were used as membrane-localized and soluble controls, respectively. The lower panel shows NSP4 VPD-WT and NSP4 VPD-Mut membrane localization. The upper panel was detected using a peptide antisera directed to NSP4 aa120–147, in the coiled-coil domain and the lower panel was detected with an anti-StrepII monoclonal antibody to detect the C-terminal epitope tags. Full-length blots are included in Supplementary Figure 3B. (**D**) Liposomes containing the fluorescent Ca^2+^ indicator Fluo-5N were suspended in HEPES buffered saline (see Materials and Methods) containing 2 mM CaCl_2_ and treated with water alone (vehicle, black) or 15 μM VPD-WT (red) or VPD-Mut (blue) dissolved in water. The change in Fluo-5N fluorescence was monitored over 20 min. The graph is a representative of 3 separate experiments. Traces are the average ± the standard deviation of 3 replicates.

To examine whether mutations of the viroporin domain disrupt NSP4 ion channel activity, we tested the function of a NSP4 mutant that substitutes aa75–80 from IFNTLL to ASDASA, which was previously shown to not elevate cytosolic calcium in mammalian cells^13^. First, we used an *E. coli* lysis assay to confirm that the VPD-Mut lacks viroporin activity (Fig. 7A). The *E. coli* lysis assay is a common method for rapidly assessing viroporin activity and was used previously to identify NSP4 mutations that disrupt viroporin function^1^. Expression of the VPD-WT protein caused *E. coli* lysis but cells expressing VPD-Mut did not lyse despite substantial protein expression (Fig. 7A,B). Western blot analysis confirmed that both VPD-WT and VPD-Mut expressed at similar rates, but the VPD-Mut construct expressed at a much higher level due to the lower cytotoxicity. These data confirm our initial findings that this mutation disrupts NSP4 viroporin activity. We previously showed that both the WT and viroporin-deficient NSP4 mutant were integral membrane proteins using a NSP4 construct consisting of both the viroporin domain and coiled-coil domain [e.g., NSP4 (47–146)]; however, since the studies presented here use the VPD alone, we sought to determine whether the mutation disrupts membrane localization of this short peptide. We expressed the VPD-WT and VPD-Mut in *E. coli*, as above, along with control NSP4 constructs known to be membrane localized [NSP4 (47–146)] or soluble [NSP4 (95–146)]. The cells were separated into soluble, peripheral, and integral membrane fractions and analyzed by immunoblotting (Fig. 7C). Similar to our previous results^13^, NSP4 (47–146) was found both in peripheral and integral membrane fractions but NSP4 (95–146) was primarily in the soluble fraction (Fig. 7C, upper panel). Both the VPD-WT and VPD-Mut peptides localized to the membrane fractions similarly to the larger NSP4 (47–146) control, confirming that the VPD is sufficient to drive membrane localization and insertion and that the mutation to disrupt viroporin activity does not alter either membrane localization or the ability to insert into the membrane.

Finally, we examined the ion channel activity of the VPD-WT and VPD-Mut peptides. Unfortunately, due to low reconstitution efficacy, we were not able to reconstitute the VPD mutant (VPD-Mut) in liposomes suitable for patch clamp. Since NSP4 is localized to the ER membrane, the ability to conduct Ca^2+^ is the most biologically relevant activity for NSP4 viroporin activity, because the ER-to-cytoplasm Ca^2+^ gradient serves as the primary electrochemical driving force across the ER membrane. Therefore, to confirm that NSP4 VPD-WT can conduct Ca^2+^ and test whether the VPD-Mut was deficient in Ca^2+^ conduction, we used a liposome-based assay that measures the fluorescence of a Ca^2+^ indicator within liposomes to determine Ca^2+^ conductivity of an ion channel^17^. Briefly, liposomes are formed in a Ca^2+^-free buffer containing Fluo-5N, a green fluorescent Ca^2+^ indicator, which is encapsulated into the liposomes. After removing the non-encapsulated dye, the liposomes were resuspended in buffer containing 2 mM CaCl_2_, baseline fluorescence measured, and 15 μM of either the VPD-WT or VPD-Mut NSP4 peptide. Ca^2+^ conducted into the liposomes by NSP4 is measured as an increase in Fluo-5N fluorescence. Addition of the vehicle alone (water) did not cause an increase in fluorescence over time (Fig. 7C, black line). Both NSP4 peptides caused an immediate increase in fluorescence due to some liposome lysis due to the localized high peptide concentration immediately after addition of the peptide. However, only VPD-WT caused a subsequent steady increase in Fluo-5N fluorescence over time (Fig. 7C, red line), while addition of the VPD-Mut peptide did not lead to increased fluorescence (Fig. 7C, blue line). This data confirmed that VPD-WT can conduct Ca^2+^ but that the VPD-Mut peptide is deficient in Ca^2+^ conductivity. Further, this assay demonstrates that NSP4 VPD-WT can conduct Ca^2+^ down a strong gradient (2 mM Ca^2+^ outside/0 mM Ca^2+^ inside) in the presence of symmetric 150 mM Na^+^, suggesting that NSP4 VPD-WT peptide can sustain a significant Ca^2+^ flux even in the presence of high monovalent cation levels, as would occur between the ER lumen and cytoplasm of RV-infected cells.

## Discussion

During a RV infection, elevated cytosolic Ca^2+^ is induced by two different forms of NSP4 that produce distinct Ca^2+^ signals. Extracellular NSP4 (eNSP4) is a secreted enterotoxin that consists of a proteolytic cleavage product of the NSP4 cytoplasmic tail and elicits a phospholipase C (PLC)-dependent Ca^2+^ flux through interaction with cell surface receptors^8^. In contrast, intracellular NSP4 (iNSP4) localizes to the ER membrane and elevates cytosolic Ca^2+^ through its viroporin activity^1^. Previous data showed that recombinant expression of iNSP4 reduced the amount of ER Ca^2+^ released upon stimulation of cells with known Ca^2+^ agonists (e.g., ATP), indicating that NSP4 increases the basal leakiness of the ER, possibly by ion channel activity^18^. These early studies were supported by the identification of the NSP4 VPD^13^,^19^ and now confirmed here by demonstrating that VPD forms a Ca^2+^-conducting ion channel.

The electrophysiology studies reported here show that the NSP4 VPD is indeed a *bona fide* ion channel, since it displays discrete levels of conductance. Interestingly, the NSP4 VPD peptide was soluble in water, which is distinct for other reported viroporins (although this may not have been tested), and we speculate that the aqueous solubility of this peptide may have contributed to the relatively low reconstitution efficiency observed. The NSP4 VPD channel was capable of conducting K^+^, Ca^2+^, and Ba^2+^ ions and shows a strong selectivity for cations over anions, but interestingly, it was not selective for Ca^2+^ over K^+^ ions. Thus, like most viroporins, NSP4 is a non-selective cation channel with stochastic gating events; however, the permeability of VPD-WT for divalent ions sets it apart from many other viroporins, which are generally monovalent cation selective with low divalent cation permeability^3^,^4^,^20^. The lack of Ca^2+^ selectivity is likely not an impediment to the release of ER Ca^2+^ in RV-infected cells because the 1000–5000 fold Ca^2+^ gradient across the ER membrane enables a sustained Ca^2+^ current even through a non-selective channel. The ability of NSP4 VPD-WT to conduct Ca^2+^ into dye-loaded liposomes in the presence of 150 mM Na^+^ supports this conclusion. These data fit with our previous observation that the ER Ca^2+^ sensor STIM1 and the store-operated calcium entry (SOCE) pathway are activated in RV-infected and NSP4-WT expressing cells, but not in cells expressing the viroporin-defective NSP4 mutant^9^, which did not conduct Ca^2+^ in the liposome assay. Thus, our data indicates that the ion channel activity of the NSP4 VPD is mechanistically responsible for the PLC-independent elevation of cytosolic Ca^2+^ by both releasing ER Ca^2+^ and through this depletion of ER Ca^2+^, inducing the activation of SOCE through the plasma membrane.

We previously demonstrated that the NSP4 viroporin-defective mutant is unable to elevate cytosolic Ca^2+^ when expressed in cells and therefore fails to induce autophagosome formation^6^,^13^. This phenotype is supported by this study that the VPD-Mut did not conduct Ca^2+^ into liposomes, which could have been caused by either a defect in VPD-Mut membrane insertion or the formation of an inactive channel complex in the membrane. However, our previous study identified the NSP4 pentalysine domain, not the amphipathic domain, as the critical motif for transmembrane insertion of NSP4^13^ and the importance of basic residues for viroporin membrane insertion was also demonstrated for the poliovirus 2B viroporin^21^. Finally, we demonstrated that the VPD-Mut construct has a similar membrane insertion profile as the VPD-WT, but since only the VPD-WT was able to conduct Ca^2+^, our data indicates that the mutant forms a defective Ca^2+^ channel complex in the membrane.

This study also showed that the kinetics of the VPD-WT is predominantly fast, with frequent transient openings. This kinetic behavior is similar to that reported for other viroporins such as HCV p7^22^ and Influenza A’s PB1-F2^17^. However, it is possible that NSP4’s ion channel characteristics may be altered or regulated by domains outside of the core viroporin domain, particularly NSP4’s cytoplasmic tail. Unlike most other viroporins, NSP4 has a long cytoplasmic coiled-coil domain (CCD) immediately downstream of the VPD that is not necessary for viroporin or ion channel activity but nevertheless may contain motifs that serve to regulate NSP4 channel activity. Recent studies have shown that the NSP4 CCD binds Ca^2+^ ions, takes on multiple oligomeric forms, and associates with several cellular and viral proteins^23^,^24^. Each of these properties offer possible mechanisms by which NSP4 channel activity could be regulated in the course of a RV infection. Of particular interest is the conserved Ca^2+^ binding site (aa120/123), which was recently shown to depend on the oligomerization state of the CCD and is regulated by changes in pH^23^,^24^. Interestingly, cellular ER-localized Ca^2+^ release channels are regulated by cytosolic Ca^2+^, but thus far, no Ca^2+^-dependent regulatory domain has been identified in a viroporin. In contrast, there is precedent for pH-mediated regulation of viroporins, since gating of the M2 viroporin from influenza A virus and the p7 viroporin from some hepatitis C virus strains are regulated by the pH of the endocytic compartment^25^,^26^. Thus, this study establishes the basis for future investigations on how NSP4 ion channel activity might be regulated through the CCD by changes in cytosolic pH or Ca^2+^ levels during the course of a RV infection.

The elevation in cytosolic Ca^2+^ by NSP4 ion channel activity is critical for productive RV replication through activation of a Ca^2+^-dependent autophagy pathway^6^. Therefore, NSP4 ion channel activity could serve as a potential drug target, since inhibition of the NSP4-mediated elevation in cytoplasmic Ca^2+^ would block RV replication. The well-defined opening events of NSP4 display a conductance in the range of ~40–60 pS similar to the conductance of L-type calcium channels, and it is possible that NSP4 could be blocked by L-type calcium channel blockers such as the benzothiazepine, dihydropyridine, or phenylalkylamine classes of blockers, which are widely used clinically, even in children^27^. Some of these drugs inhibit Ca^2+^ influx into RV-infected cells, which was predicted to be through blocking plasma membrane-localized host Ca^2+^ channels; however, it is possible these drugs may also inhibit NSP4 functions^28^. Further, blockers of NSP4 ion channel activity may also potently block RV-induced secretory diarrhea, because the NSP4-mediated elevation in cytoplasmic Ca^2+^ is critical for Cl^−^ secretion both from RV-infected enterocytes and through activation of the enteric nervous system *via* increased serotonin secretion^29^,^30^.

The best characterized viroporins that affect cellular Ca^2+^ levels are RV NSP4 and enterovirus 2B, and thus far their effects on Ca^2+^ levels have been characterized using fluorescent Ca^2+^ imaging techniques^1^. While both NSP4 and 2B are widely considered putative ion channels, this electrophysiology study demonstrates directly that NSP4 has *bona fide* ion channel activity and can conduct Ca^2+^. Recent studies show that other viroporins, previously characterized as being monovalent cation selective, can also conduct Ca^2+^ under some conditions. The E protein of severe acute respiratory syndrome (SARS) coronavirus can conduct Ca^2+^ when reconstituted into membranes that mimicked those of the ERGIC/Golgi compartments^31^. This opens the possibility that other viroporins, previously characterized to be monovalent cation selective, may support Ca^2+^ conductivity under the right experimental conditions, and exploitation of host Ca^2+^ signaling pathways by viroporin activity may be more common than currently appreciated.

## Additional Information

**How to cite this article**: Pham, T. *et al*. The Rotavirus NSP4 Viroporin Domain is a Calcium-Conducting Ion Channel. *Sci. Rep.* **7**, 43487; doi: 10.1038/srep43487 (2017).

**Publisher's note:** Springer Nature remains neutral with regard to jurisdictional claims in published maps and institutional affiliations.

## Supplementary Material

Supplementary Data

## Figures and Tables

**Table 1:** Reconstitution efficiency and kinetic distribution from patches studied with the dehydration/rehydration method (DHRH) vs. the cloud method.

DHRH		Clouds	
Empty Patches	240	Empty Patches	250
Well-defined only	11	Well-defined only	7
Fast kinetics only	10	Fast kinetics only	7
Mixed	7	Mixed	12
Total Patches	268	Total Patches	276
Reconstitution Efficiency	10.45%	Reconstitution Efficiency	9.42%

The tables gives the number of patches with either no channels (“empty patches”), or with channels displaying well-defined openings only or fast kinetics only (illustrated in Fig. 2), or a mixture of both behaviors (“Mixed”, as illustrated in Fig. 3). A total of 544 patches were obtained with reconstituted VPD-WT in the course of this study. The reconstitution efficiency was calculated by dividing the number of patches with activity by the total number of patches.
