# Supplementary material for: The Rotavirus NSP4 Viroporin Domain is a Calcium-conducting Ion Channel
Source: Sci Rep. 2017 Mar 3;7:43487. doi: 10.1038/srep43487 (PMC5335360; doi:10.1038/srep43487)
Supplement: Supplementary Data [file srep43487-s1.pdf]

The Rotavirus NSP4 Viroporin Domain is a Calcium-conducting Ion Channel

(Supplemental Data)

Thieng Pham[1], Jacob L. Perry[2], Timothy L. Dosey[3], Anne H. Delcour[1], Joseph Hyser[2*]

[1]Department of Biology and Biochemistry, University of Houston, Houston, TX, [2]Alkek Center for Metagenomic and Microbiome Research, Department of Molecular Virology and Microbiology, [3]Verna and Marrs McLean Department of Biochemistry and Molecular Biology, Baylor College of Medicine, Houston, TX.

Supplemental Figure 1

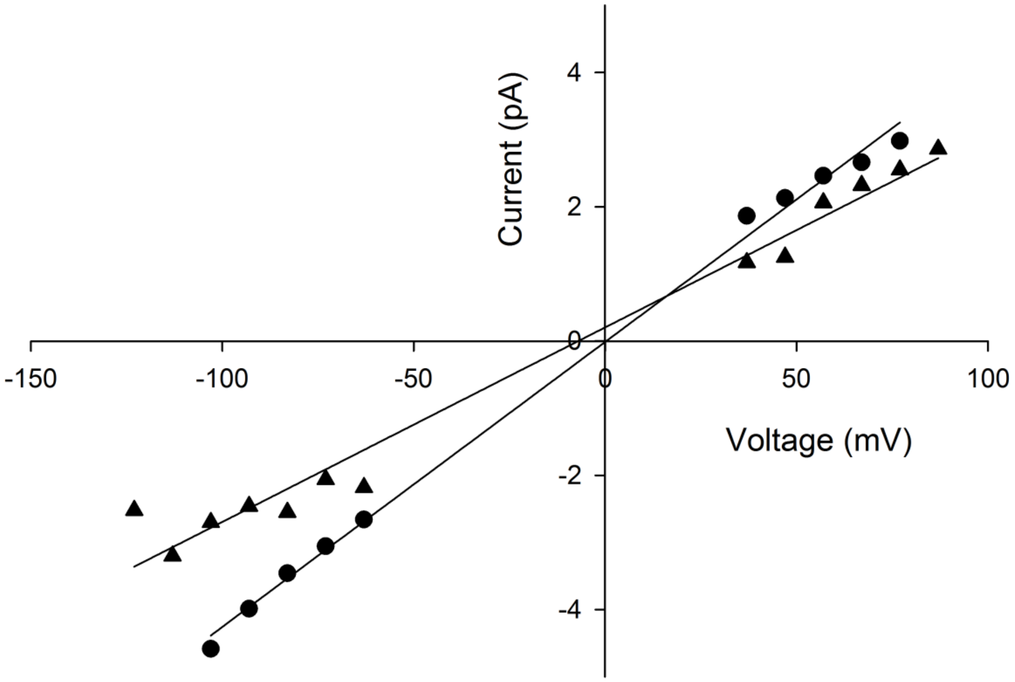

Supplemental Figure 1. Current-voltage relationships of VPD-WT. The currents were measured from well-defined openings at several voltages in symmetric buffer A (circles) and asymmetric buffer A/buffer CA (triangles) solutions. There was a ~13 mV offset potential in symmetric buffer A. Therefore all membrane potentials were corrected by the same offset in symmetric and asymmetric conditions. The lines represent the linear regressions through the data points, and the slopes give calculated conductances of 42 pS in symmetric buffer A and 29 pS in buffer A/CA.

Supplemental Figure 2

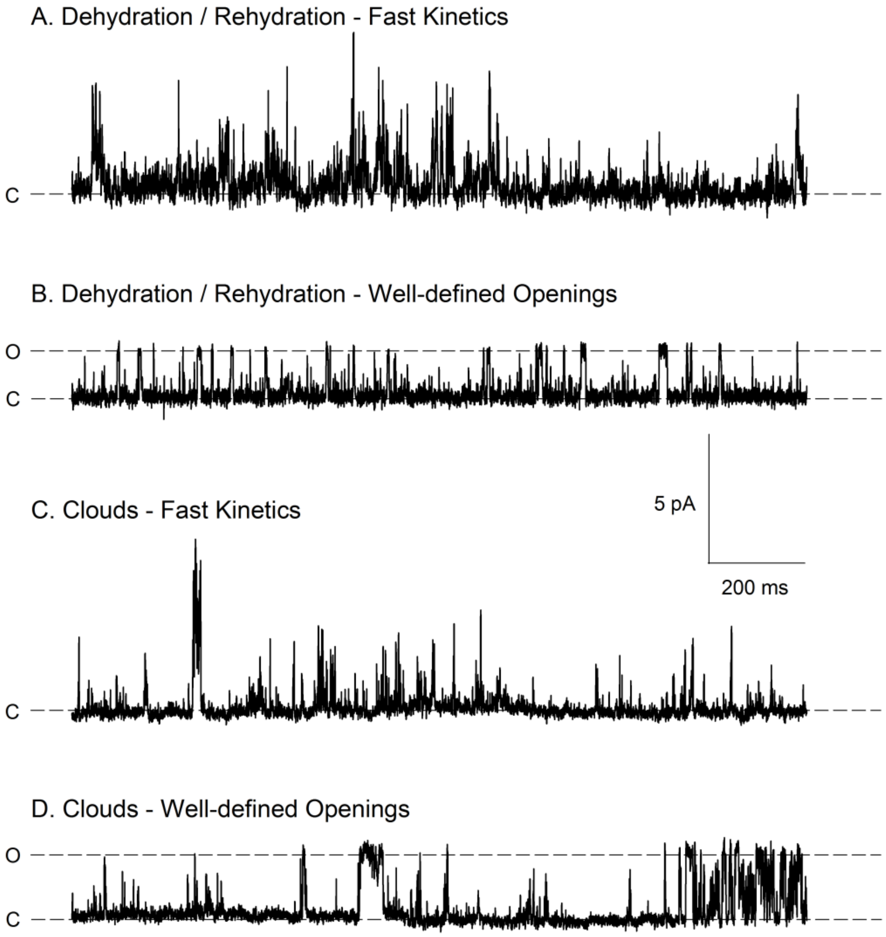

Supplemental Figure 2. Similar kinetic signatures were observed regardless of reconstitution method. VPD-WT peptide was dissolved in DMSO and reconstituted either with the dehydration-rehydration method at protein:lipid ratios (w:w) of 1:3250 (A) or 1:3000 (B), or with the cloud method at a protein:lipid ratio (w:w) of 1:200 (C-D). The traces were obtained at +50 mV in symmetric buffer A. The conductance of the open state was 35 pS (B) and 49 pS (D). In A & C, the conductance could not be determined due to a lack of a defined current level of the open state.

Supplemental Figure 3

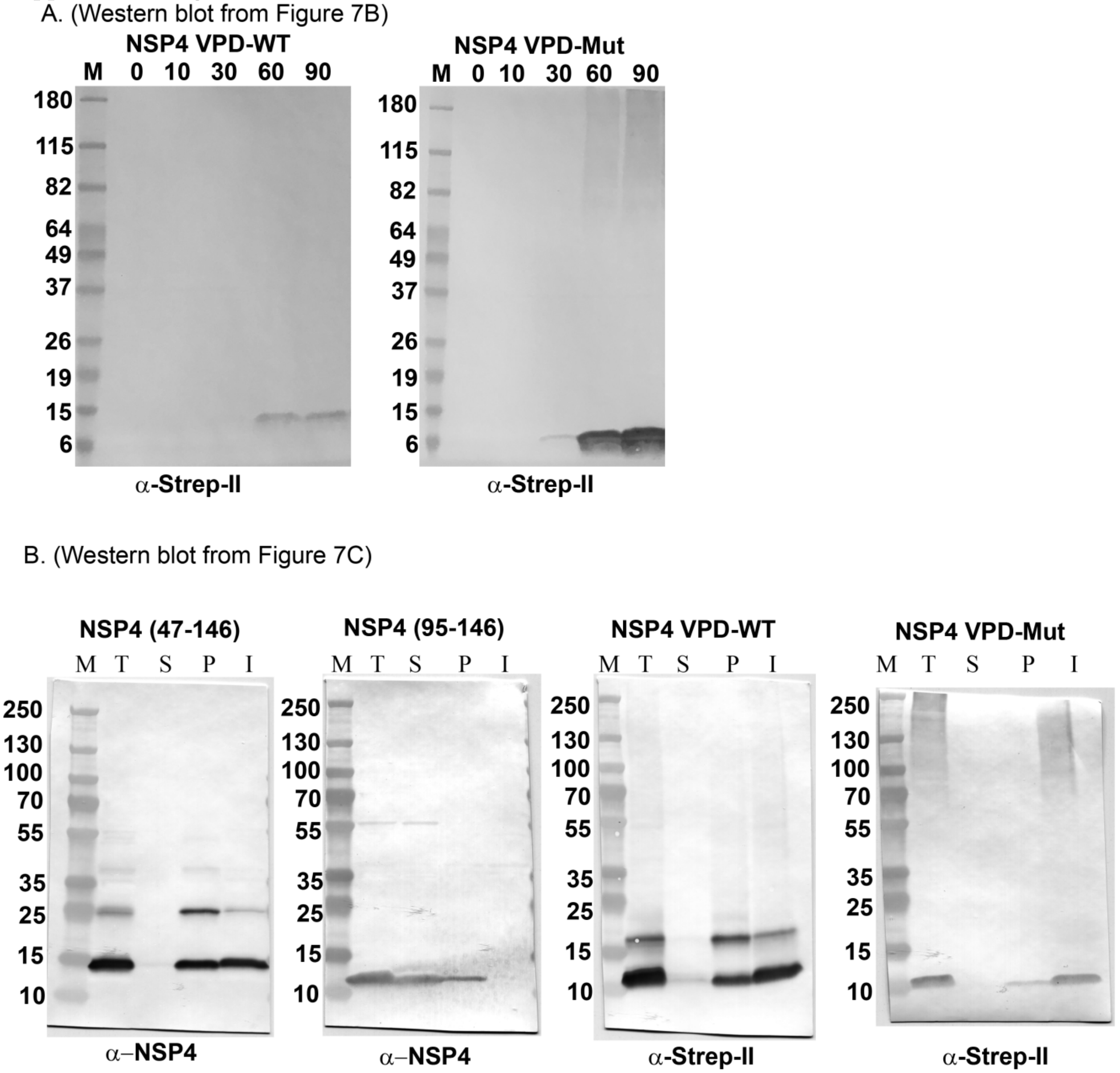

Supplemental Figure 3. Full size western blots from Figure 7. (A) Western blot analysis of bacterial lysis assay samples of time points 0, 10, 30, 60, and 90 from NSP4VPD-WT and NSP4 VPD-Mut. Mouse anti-StrepII tag antibody was used for primary antibody staining. These blots correspond to Figure 7B in the main paper. (B) Western blot analysis for integral membrane localization of the NSP4 viroporin domain. Rabbit anti-NSP4 (120-147) was used for primary antibody staining of NSP4 (47-146) and NSP4 (95-146), and Mouse anti-StrepII tag antibody was used for primary antibody staining of NSP4 VPD-WT, and NSP4 VPD-Mut. Lane labels are: Molecular weight marker (M), Total protein (T), Soluble proteins (S), Peripheral Membrane (P), and Integral Membrane (I). These blots correspond to Figure 7C in the main paper.
